# Functional characterization of Mousterian tools from the Caucasus using comprehensive use-wear and residue analysis

**DOI:** 10.1038/s41598-022-20612-x

**Published:** 2022-10-19

**Authors:** E. V. Doronicheva, L. V. Golovanova, J. V. Kostina, S. A. Legkov, G. N. Poplevko, E. I. Revina, O. Y. Rusakova, V. B. Doronichev

**Affiliations:** 1 ANO Laboratory of Prehistory, Liflyandskaya street 6M, St. Petersburg, Russia 190020; 2 A.V. Topchiev Institute of Petrochemical Synthesis, Russian Academy of Sciences, Leninskiy prospekt 29-2, Moscow, Russia 119991; 3 Laboratory for Experimental–Traceological Studies, Institute for the History of Material Culture, Russian Academy of Sciences, Dvortsovaya embankment 18, St. Petersburg, Russia 191186; 4 Rostov Regional Museum of Local Lore, Bolshaya Sadovaya street 79, Rostov-on-Don, Russia 344006

**Keywords:** Anthropology, Archaeology, Cultural evolution, Organic chemistry

## Abstract

The authors discuss functional characterization of Mousterian tools on the basis of their use-wear and residue analysis of five lithic tools from Mezmaiskaya cave and Saradj-Chuko grotto in the North Caucasus. The results represent the first comprehensive use-wear and residue analysis carried out on Mousterian stone artefacts in the Caucasus. This study unequivocally confirms the use of bitumen for hafting stone tools in two different Middle Paleolithic cultural contexts defined in the Caucasus, Eastern Micoquian and Zagros Mousterian.

## Introduction

The development of composite technology using adhesive materials is often seen as a hallmark of cognitive sophistication that played an important role in the social and technological development of the genus *Homo* [e.g.,^1,2^]. Our understanding of the use of composite tools by the Middle Palaeolithic (MP) Neanderthals in Eurasia relies on evidence of hafting and adhesives^3,4^. Most ideas on the development of Palaeolithic composite tool technologies are based on microscopic use-wear, including diagnostic impact fractures (DIFs) and other traces of use^5–9,11–15^, and diagnostic characteristics of hafting traces^10^ (further, diagnostic hafting traces, DHTs), and the morphology of tools (i.e., the presence of hafting elements). However, the exact hafting significance of use-wear traces and morphological features is not always clear^16^, and this evidence alone are not an exhausted indication of the presence of hafting technology. Also, some studies indicate that the interpretative potential of some impact fractures proposed as having diagnostic value for the identification of projectiles is still unclear^17,18^.

The lithic residue analysis provides direct information that the lithic artefacts were hafted, as well as allows precise identifying the adhesive materials involved in the manufacture of these composite tools. The currently known unambiguous evidence of the securely dated, and chemically and spectrometrically identified MP hafting adhesives includes three flakes with birch tar from Campitello Quarry (Italy) and Zandmotor (Netherlands)^19,20^, two lumps of birch tar that were probably attached to a bifacial knife from Königsaue (Germany)^21^, and nine tools and flakes with pine resin, and one scraper with pine resin and beeswax from Fossellone and Sant’Agostino caves (Italy)^22^ in Europe, as well as 14 tools and flakes with bitumen from the sites of Umm El Tlel and Hummal (Syria) in the Levant^23–26^. These studies document that adhesive technology was used in both Europe and south-west Asia by varied Neandertal populations and the MP production of adhesives was complex. Neandertals mixed pine resin with beeswax^22^ and bitumen with quartz and gypsum^24^, and distilled tar from birch bark^20^.

Despite the MP adhesive evidence is being increasingly documented in Europe and Asia [for modern review see^20^], the level of adhesive technology applied for manufacturing composite tools among different Neanderthal groups is problematic given the lack of relevant data from the majority of MP regional contexts. This state of research demonstrates the need for detailed modern studies about the role of adhesives in hafting and the level of hafting technology in various MP regions.

**Figure 1:**
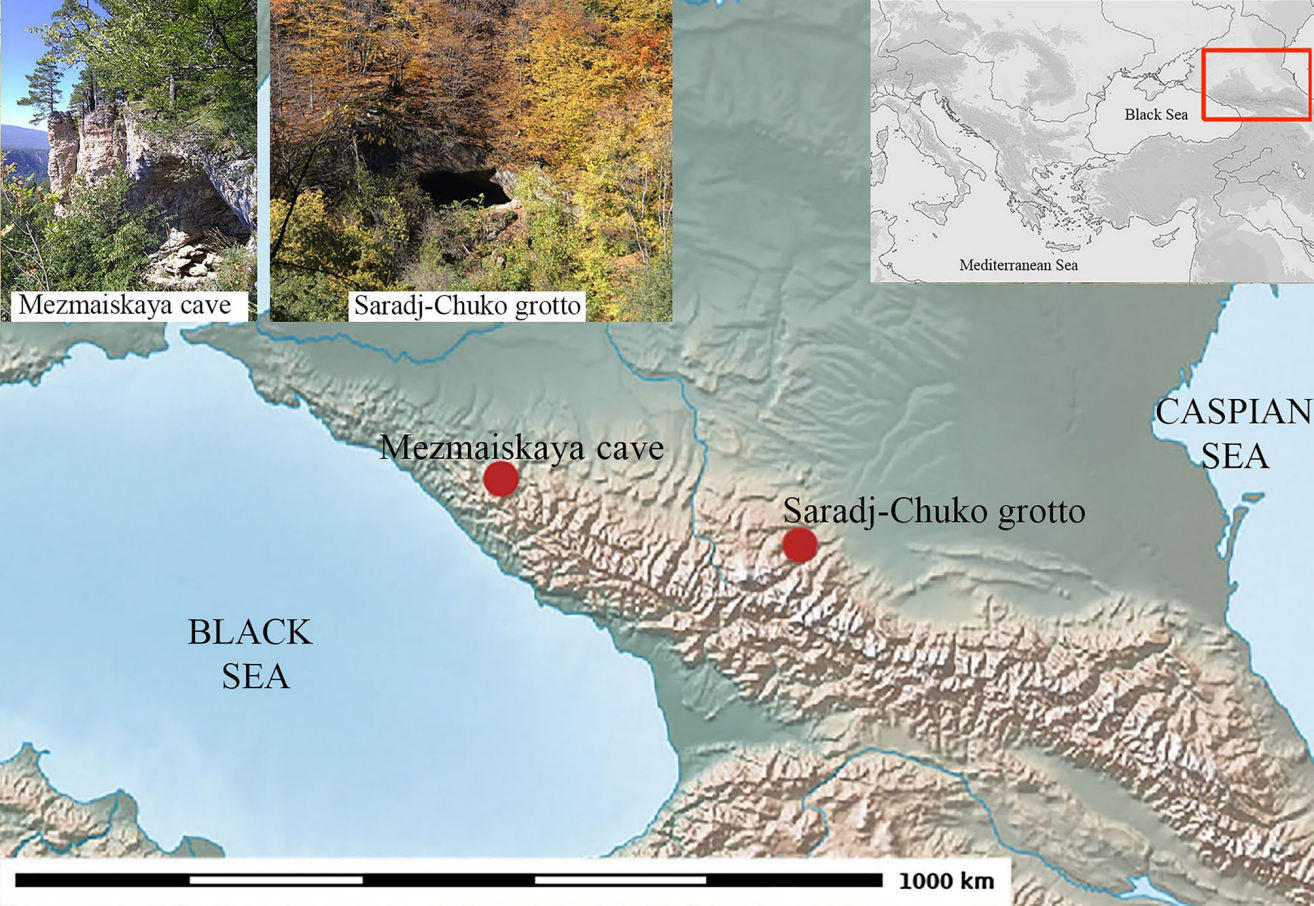
Map of the Caucasus showing the location of Mezmaiskaya cave and Saradj-Chuko grotto. Data: Natural Earth. Figure produced using GRASS GIS 7.8 and Inkscape 0.97.

Our case study is a sample of five lithic tools (Table 1) that were recovered from modern excavations in MP levels at Mezmaiskaya cave and Saradj-Chuko grotto in the North Caucasus^27,28^ (Fig. 1). The results reported in this paper represent the first comprehensive use-wear and residue analysis carried out for MP artefacts in the Caucasus. This study unequivocally confirms the use of bitumen for hafting stone tools in two different MP cultural contexts in the Caucasus, Eastern Micoquian and Zagros Mousterian.

**Table 1:** Detail identification of the archaeological samples analyzed.

Sample No	Barcode-ID number	Dimensions, in cm (length, width, thickness)	Typological definition; raw material	Functional identification
1	Saradj-Chuko, 2018, 6A, hor. 2, #1	3.8 × 3.4 × 1.1	Truncated-faceted side-scraper; gray flint	Meat knife
2	Saradj-Chuko, 2018, 6B, hor.2, #193	4.7 × 2.8 × 0.9	Mousterian point; obsidian with black and red-brown inclusions	Projectile tip
3	Saradj-Chuko, 6B, hor. 2, #502	4.8 × 4.3 × 0.9	Convergent scraper with a truncated-faceted base; light gray flint	Projectile tip/meat knife
4	Saradj-Chuko, 6B, hor. 3, #266	4.4 × 3.4 × 1.3	Convergent scraper with thinned base; pinkish-gray flint	Projectile tip/meat knife
5	Mezmaiskaya, 2B-3, square N-24, #25/571	3.6 × 3.7 × 0.8	Convergent scraper; light gray flint	Projectile tip/meat knife

## Results

### Use-wear and hafting identification

We used reflected light stereomicroscopy (with magnification < 100 ×) for identifying traces related to use-wear and hafting on the analyzed lithic tools. Also, reflected light stereomicroscopy was used initially to localize identifiable residues on the tools.

**Figure 2:**
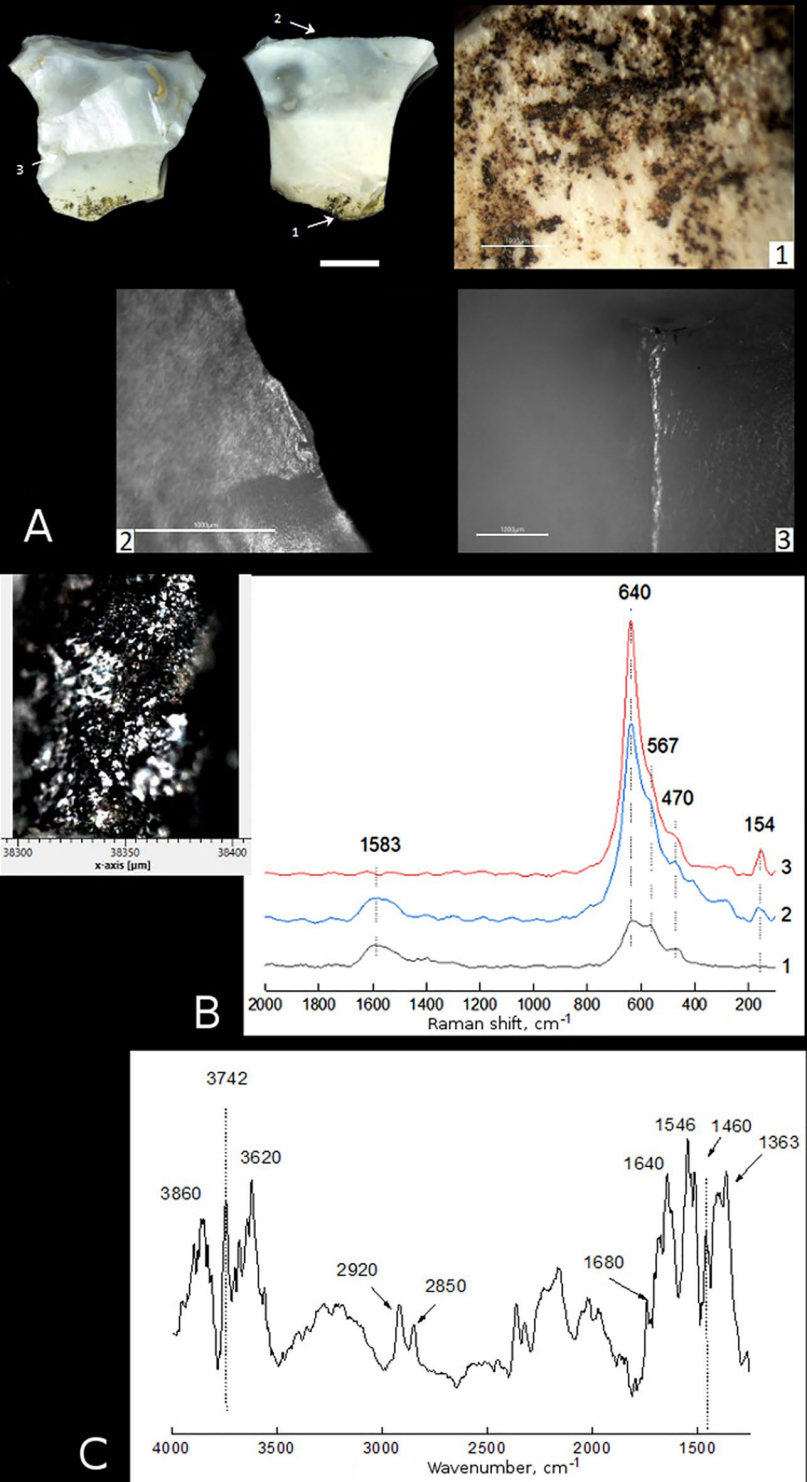
Use-wear and residue analyses of sample 1 from Layer 6A at Saradj-Chuko grotto. (**A**) Photo of sample 1, scale bar = 1 cm. The arrows indicate position of close-ups of residues and use-wear traces shown on microscopic photos 1–3, scale bar = 1000 μm: (1) residues of black substance (bitumen) on the tool base, (2) polishing and smoothing of the tool working edge due to use, (3) polishing and smoothing of a scar ridge on the dorsal surface due to the tool hafting. (**B**) SEM close-up on the sample of black substance (bitumen residue) analyzed by FTIR and Raman spectroscopy, and Raman spectra of three residue samples. (**C**) FTIR spectrum of the same residue.

**Figure 3:**
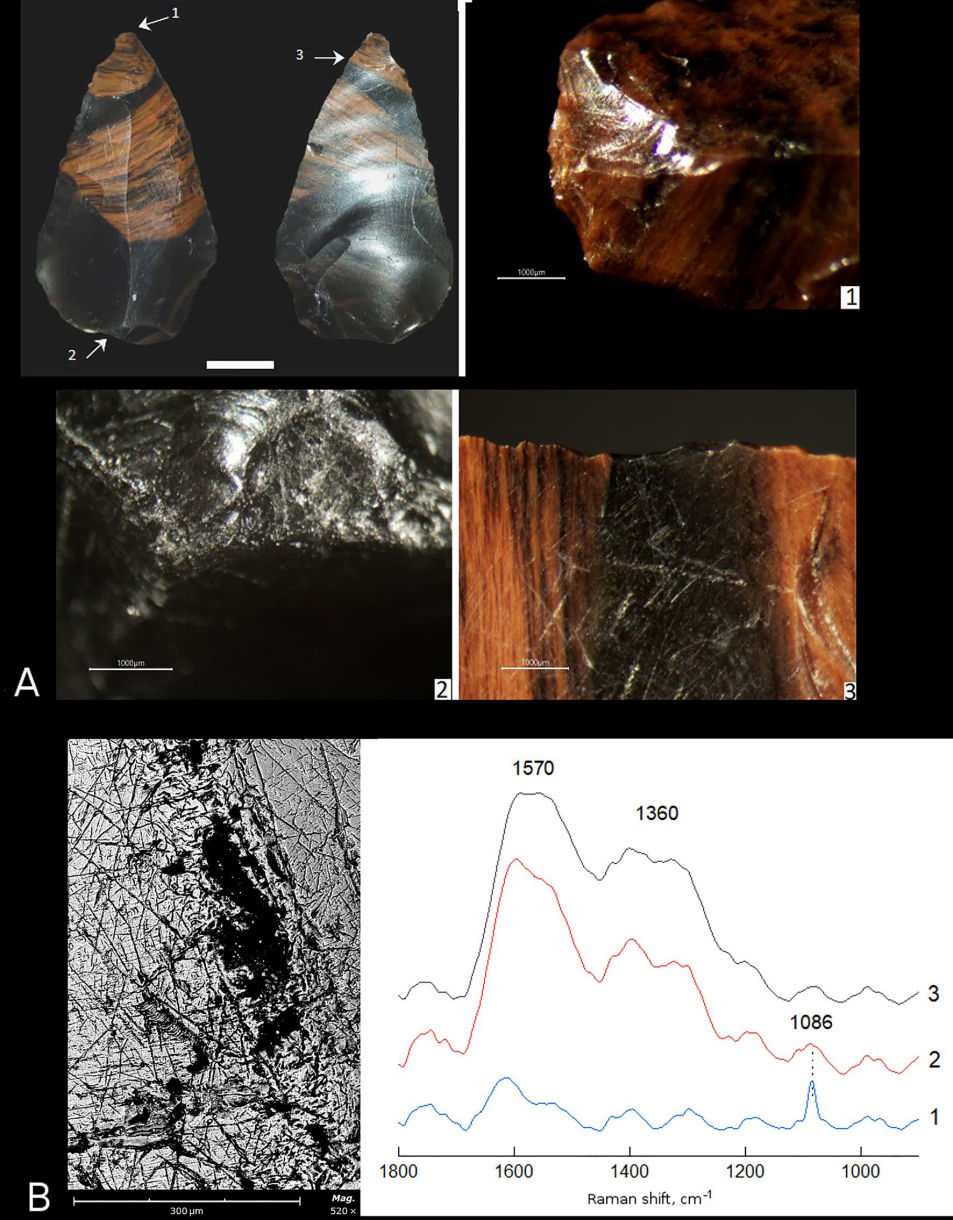
Use-wear and residue analyses of sample 2 from Layer 6B at Saradj-Chuko grotto. (**A**) Photo of sample 2, scale bar = 1 cm. The arrows indicate position of close-ups of use-wear traces shown on microscopic photos 1–3, scale bar = 1000 μm: (1) fractures on the tip, (2) crushing area on the tool base, (3) smoothing and linear striations along the tool edge. (**B**) SEM close-up on a sample of the black substance (bitumen residue) analyzed by FTIR and Raman spectroscopy, and Raman spectra of three residue samples.

Sample 1 is a truncated-faceted side-scraper. The upper and right sides of the tool are worked by abrupt and semi-abrupt, multi-row retouch from the dorsal surface. On the ventral surface along the upper edge there are areas of irregular micro-retouch and bright polishing on protruding ridges (Fig. 2A-2). These features are typical for so called 'meat polish', indicating the tool was used for cutting meat. The left side is worked using the truncating-faceting method. The edge is partially rounded, and exhibits areas of abrasion and polishing on protruding ridges, which is typical for so called 'wood polish'. Also, similar polishing is observed on protruding ridges on the dorsal surface near the tool base (Fig. 2A-3). These are DHTs related to the tool contact (friction and motion) with a haft^29,30^, and indicating the left side and the base of the tool were hafted, likely in a wooden haft. The use-wear and hafting traces identified on the tool suggest that it was used as a meat knife that was hafted. This conclusion is confirmed by the presence of numerous residues of a dark brown, locally black substance morphologically similar to bitumen from both the dorsal and ventral surfaces on the tool base (Fig. 2A-1).

Sample 2 is an elongated Mousterian point. The right and left convergent sides are worked with abrupt and semi-abrupt retouch from the dorsal surface. From the dorsal surface along both converging edges there are numerous micro-fractures, as well as areas of mainly single-row and rarer two-row micro-retouch on protruding ridges. The tip is broken, and the preserved portion of the tip shows a transverse bending fracture with small spin-offs along the ridge created by primary fracturing on the dorsal surface (Fig. 3A-1). These are DIFs fractures, which provide proxies to indicate potential use of the lithic point as a tip of composite projectile. Light smoothing and rounding, and linear parallel striations going from the edge are observed on the ventral surface along the right edge near the broken tip (Fig. 3A-3). The edge of the tool base shows numerous crushing areas and micro-fractures (Fig. 3A-2), as well as several abrasion areas from the ventral surface, suggesting the tool was hafted. The DIFs and hafting traces identified on the tip and base of the tool respectively suggest that the lithic point was used as a projectile tip that was mounted on a shaft. This proposal is confirmed by the presence of micro-residues of a black substance morphologically similar to bitumen on several areas along the edge of the tool base (Fig. 3B).

**Figure 4:**
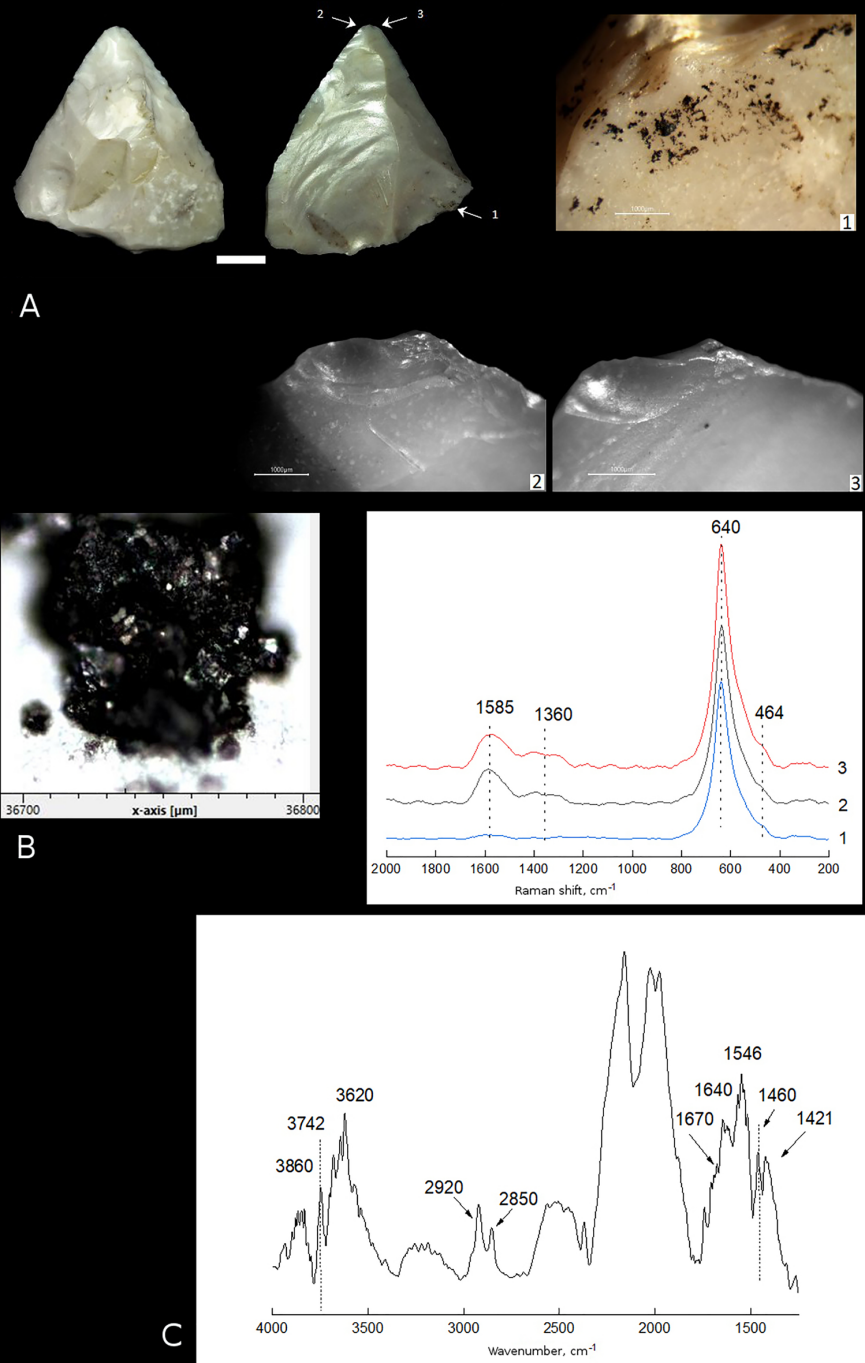
Use-wear and residue analyses of sample 3 found in Layer 6B at Saradj-Chuko grotto. (**A**) Photo of sample 3, scale bar = 1 cm. The arrows indicate position of close-ups of residues and use-wear traces shown on microscopic photos 1–3, scale bar = 1000 μm: (1) residues of black substance (bitumen) on the tool base, (2, 3) polishing and spin-off fractures on the tip. (**B**) SEM close-up on a sample of the black substance (bitumen residue) analyzed by FTIR and Raman spectroscopy, and Raman spectra of three residue samples. (**C**) FTIR spectrum of the same residue.

Sample 3 is a convergent scraper with a truncated-faceted base. The right and partially left convergent sides of the tool, and partially the tool base are worked with abrupt retouch from the dorsal surface. The tool convergent edges are smooth in plan view and finely denticulated in profile, with micro-fractures along the edges. The dorsal surface was thinned from the tool base using the truncating-faceting method. The tool tip is broken and the preserved portion of the tip shows DIFs, such as small spin-off fractures from the ventral and dorsal surfaces, which initiate from the same bending fracture that removed the tip (Fig. 4A-2,3). On both right and left edges of the tool near the tip there are spots of bright polishing with a greasy sheen, which is going mainly along protruding ridges, partially smoothing the edges, and not spreading over the tool surfaces, which is typical for meat polish. The tool base has areas of bright (‘mirror type’) polishing that is partially covering the retouch facets and extending far from the base edge, which is typical for wood or antler hafting polish^31^. These DHTs suggest the tool base was hafted in a wooden/antler haft. The use-wear and hafting traces, and DIFs identified on the tool suggest the convergent scraper was used as a projectile tip or meat knife, and that the tool was probably hafted. This proposal is confirmed by the presence of micro-residues of a dark brown, locally black substance morphologically similar to bitumen on several areas along the tool base (Fig. 4A-1).

**Figure 5:**
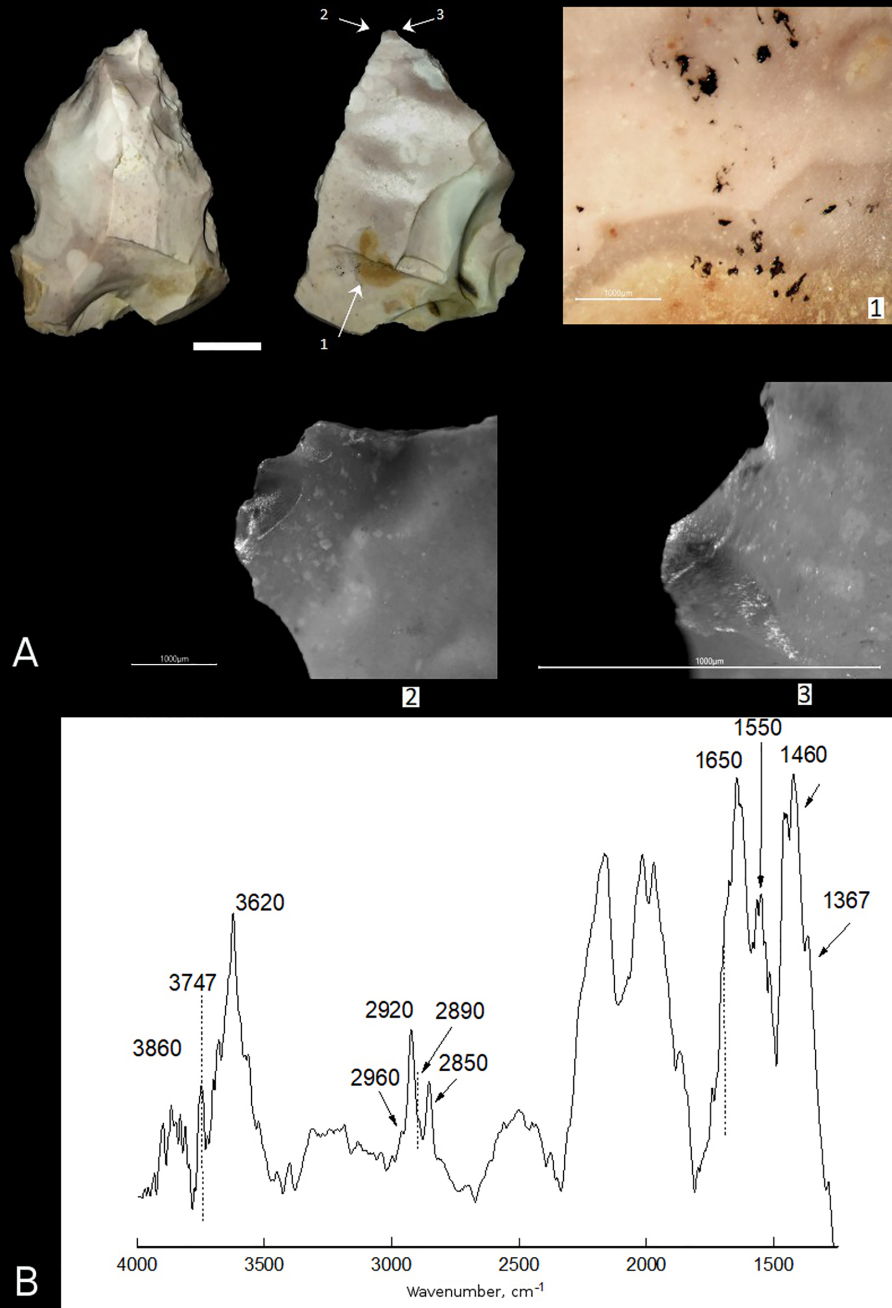
Use-wear and residue analyses of sample 4 found in Layer 6B at Saradj-Chuko grotto. (**A**) Photo of sample 4, scale bar = 1 cm. The arrows indicate position of close-ups of residues and use-wear traces shown on microscopic photos 1–3, scale bar = 1000 μm: (1) residues of black substance (bitumen) on the tool base, (2, 3) (2, 3) polishing and spin-off fractures on the tip. (**B**) FTIR spectrum of the black substance (bitumen residue) found on sample 4.

Sample 4 is a convergent scraper with thinned base. The right and partially left convergent sides of the tool are worked with abrupt retouch from the dorsal surface. The tool convergent edges are smooth in plan view and finely denticulated in profile, with micro-fractures along the edges. The basal part of the tool is thinned from the ventral surface by flat scars going from the tool base. The tool tip has small spin-off fractures on the ventral and dorsal surfaces (Fig. 5A-2,3) that represent DIFs. Along the tool edges there are spots of bright polishing with a greasy sheen, which is going mainly along protruding ridges, partially smoothing the edges, and not spreading over the tool surfaces, which is typical for meat polish. The tool base shows bright, 'mirror type' polishing, forming continuous strips on some areas and extending far from the base edge, which is typical for wood hafting polish. This suggests the tool base was hafted in a wooden haft. The use-wear and hafting traces, and DIFs identified on the tool suggest the convergent scraper was used originally as a projectile tip and secondary as a meat knife, and that the tool was hafted. The tool hafting is confirmed by the presence of micro-residues of a dark brown, locally black substance morphologically similar to bitumen on the tool base from the ventral surface (Fig. 5A-1).

**Figure 6:**
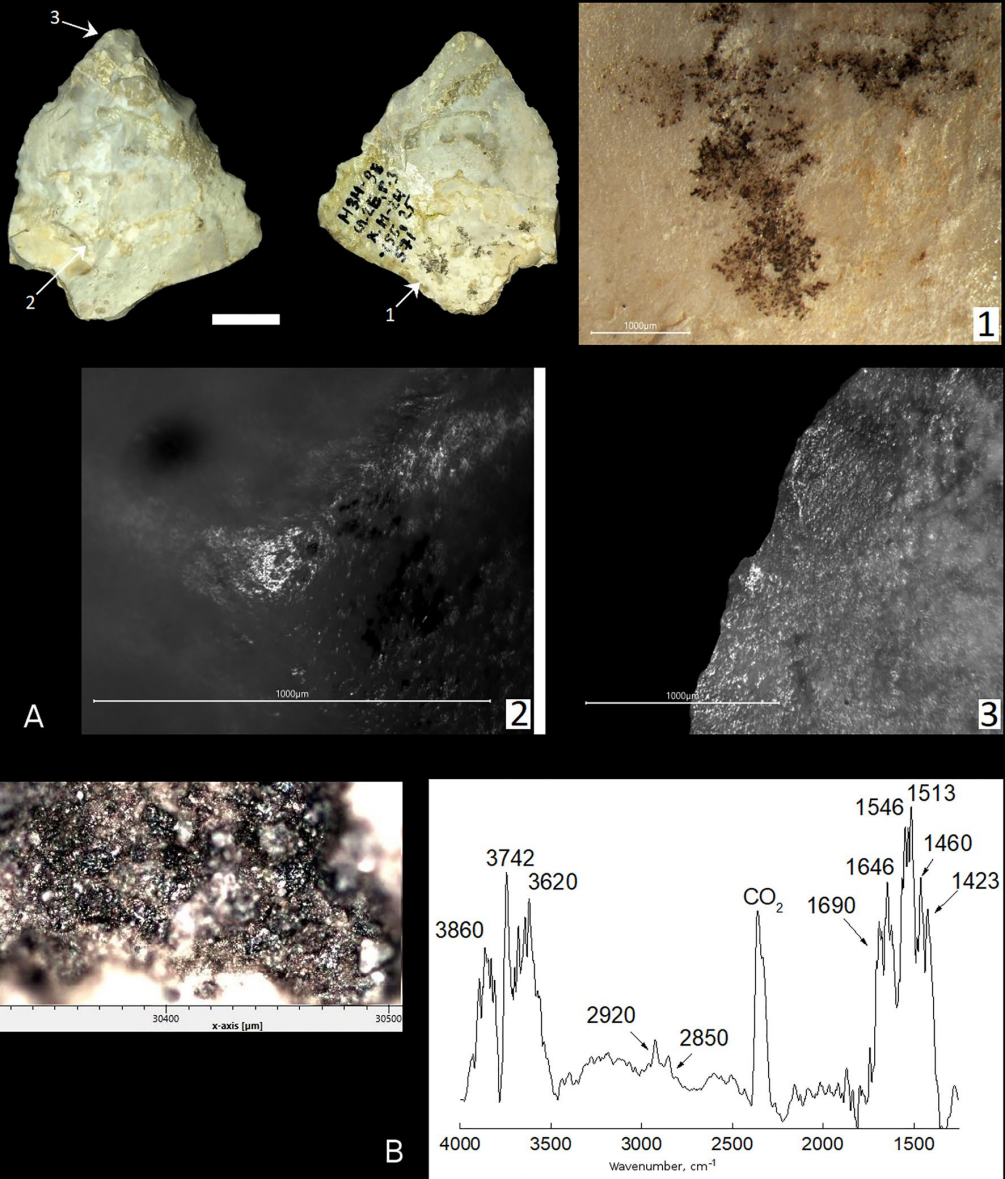
Use-wear and residue analyses of sample 5 found in Layer 2B-3 at Mezmaiskaya cave. (**A**) Photo of sample 5, scale bar = 1 cm. The arrows indicate position of close-ups of residues and use-wear traces shown on microscopic photos 1–3, scale bar = 1000 μm: (1) residues of black substance (bitumen) on the tool base, (2, 3) polishing and fractures on the tip. (**B**) SEM close-up on a sample of the black substance (bitumen residue) analyzed by FTIR and Raman spectroscopy, and a FTIR spectrum of the residue.

Sample 5 is a convergent scraper. From the dorsal surface, the right side of the tool is worked with abrupt retouch, with micro-fractures along the edge, and the left side is partially worked with small, semi-abrupt retouch. The tool edges on both convergent sides are smooth in plan view and finely denticulated in profile. The tool tip has small spin-off fractures on the ventral and dorsal surfaces that represent DIFs. Along the left tool edge and especially on the tool edge near the tip there is spotty bright polishing with a greasy sheen. The polishing covers mainly protruding areas along the edge, partially smoothing retouch facets and the edge, and not spreads over the tool surface, which is typical for meat polish (Fig. 6A-3). The right tool edge has not such evidence of intensive use of the tool as a meat knife. In the basal part of the tool there are areas of bright polishing, concentrating mainly on protruding ridges and forming continuous strips on some areas. The polishing covers microrelief of the surface and extends over the base of the tool, which is typical for wood hafting polish (Fig. 6A-2). This suggests the tool base was hafted in a wooden haft. The use-wear and hafting traces, and DIFs identified on the tool suggest the convergent scraper was used as a projectile tip or meat knife, and that the tool was probably hafted. We did not identified any post-depositional alterations, such as soil polish (often with micro-striations from sand particles), on this tool, except the calcite-carbonate crust on some areas of the tool surface, which looks fresh in all areas not covered with the crust. The tool hafting is confirmed by the presence of micro-residues of a dark brown, locally black substance morphologically similar to bitumen on the tool base from the ventral surface (Fig. 6A-1).

### Adhesive identification

Possible bitumen residues have been identified on all five lithic tools analyzed in this study. For the precise identification of the residues as the organic bitumen, each sample was studied using three methods, including Fourier transform infra-red (FTIR) spectroscopy, Raman spectroscopy, and scanning electron microscopy with energy dispersive spectroscopy (SEM–EDS). In our approach, SEM imaging (with magnification > 100 ×) was used for a detailed visualization of the residues. SEM–EDS, FTIR and Raman spectroscopy were used to yield chemical and vibration spectroscopic data. The FTIR and Raman spectroscopic techniques defined absorption bands indicative for organic bitumen on the analyzed archaeological samples. SEM–EDS were used to identify main chemical elements and compare the elemental composition of bitumen residues on different archaeological samples.

#### FTIR and Raman spectroscopy

The FTIR and Raman spectroscopy results indicate the presence of the absorption bands corresponding to specific bitumen bands^32–36^ in all residues identified on the analyzed samples. The results of FTIR and Raman spectroscopy of archaeological residues on samples 1–5 are summarized in Table 2.

**Table 2:** Values of wavenumber and Raman shift indicative for the identification of bitumen^32–36^, which were defined in residues on archaeological samples 1–5 on the basis of FTIR and Raman spectroscopy respectively.

Values of Wavenumber or Raman Band Shift [cm^–1^]	Description of the Band Assignment
**Sample 1**	
2920	Asymmetric stretching vibrations ν (C–H) in CH_2_– group
2850	Symmetric stretching vibrations ν (C–H) in CH_2_– group
1460	Deformational vibrations in CH– group
1363	Deformational vibrations in CH– group
1583	**Raman peak G (around 1600 cm**^**−1**^**)**, vibration within the aromatic ring in the graphene cluster
**Sample 2**	
1570	**Raman peak G (around 1600 cm**^**−1**^**), vibration within the aromatic ring in a graphene-like cluster**
1360	**Raman peak D (around 1350 cm**^**−1**^**), vibration within the aromatic ring in the graphene cluster**
**Sample 3**	
2920	Asymmetric stretching vibrations ν (C–H) in CH_2_– group
2850	Symmetric stretching vibrations ν (C–H) in CH_2_– group
1460	Deformational vibrations in CH– group
1421	Deformational vibrations in CH– group
1585	**Raman peak G (around 1600 cm**^**−1**^**), vibration within the aromatic ring in the graphene cluster**
1360	**Raman peak D (around 1350 cm**^**−1**^**), vibration within the aromatic ring in the graphene cluster**
**Sample 4**	
2960	Asymmetric stretching vibrations ν (C–H) in CH_3_– group
2920	Asymmetric stretching vibrations ν (C–H) in CH_2_– group
2890	Symmetric stretching vibrations ν (C–H) in CH_3_– group
2850	Symmetric stretching vibrations ν (C–H) in CH_2_– group
1460	Deformational vibrations in CH– group
1367	Deformational vibrations in CH– group
**Sample 5**	
2920	Asymmetric stretching vibrations ν (C–H) in CH_2_– group
2850	Symmetric stretching vibrations ν (C–H) in CH_2_– group
1460	Deformational vibrations in CH– group
1423	Deformational vibrations in CH– group

Raman band assignments are in bold.

Sample 1. At the high magnification (> 100 ×), the residues preserved on this sample (Fig. 2A-1) appear black in color (Fig. 2B). The FTIR spectrum of the residue (Fig. 2C) indicates specific bitumen bands, such as the bands at 2920 and 2850 cm^−1^ corresponding to asymmetric and symmetric stretching vibrations ν (C–H) in CH_2_– group (methylene), and the bands at 1460 and 1363 cm^−1^ corresponding to deformational vibrations of CH– group. The absorption bands at 1680 and 1546 cm^−1^ additionally confirm the presence of organic matter in the residue, but are not diagnostic for the identification of bitumen. Raman spectra of two of the three analyzed in total samples of the same residue (Fig. 2B) show the band at 1583 cm^−1^, which corresponds to the Raman peak G reflecting vibrations within the aromatic ring of the graphene cluster characteristic of bituminous mixtures. However, all three spectra lack the absorption bands corresponding to the Raman peak D (around 1340–1360 cm^−1^), which is also typical to graphene.

Sample 2. FTIR spectroscopy of the residue on this sample failed, as all FTIR spectra reflected only a solid background noise. Raman spectra of two of the three analyzed in total samples of the same residue (Fig. 3B) show wide bands at 1570 and 1360 cm^−1^, which correspond to Raman peaks G and D (around 1600 cm^−1^ and around 1350 cm^−1^ respectively; ^36^: tab. 2), indicating the presence of graphene or graphitic components that are typical to bitumen.

Sample 3. At the high magnification (> 100×), the residues preserved on this sample (Fig. 4A-1) appear black in color (Fig. 4B). The FTIR spectrum of the residue on sample 3 (Fig. 4C) is similar to the FTIR spectrum of the residue on sample 1. Like sample 1, the FTIR spectrum of the residue on sample 3 shows the bands at 2920 and 2850 cm^−1^ (stretching vibrations ν (C–H) in CH_2_– group), and the bands at 1460 and 1421 cm^−1^ (deformational vibrations in CH– group) that are typical to organic bitumen, as well as the bands at 1670 and 1546 cm^−1^ confirming the presence of organic matter in the residue. Raman spectra of two of the three analyzed in total samples of the same residue (Fig. 4B) show bands at 1585 and 1360 cm^−1^, which correspond to Raman peaks G and D. Similar bands characteristic of the graphene component, which is typical to bitumen, were identified also in sample 2.

Sample 4. The FTIR spectrum (Fig. 5B) of the residue on this sample (Fig. 5A-1) is similar to the FTIR spectra of the residues on samples 1 and 3. In comparison to samples 1 and 3, the FTIR spectrum of sample 4 shows the highest diversity of absorption bands that are typical to organic bitumen, including the bands at 2920 and 2850 cm ^−1^ (vibrations ν (C–H) in CH_2_– methylene group), small peaks at 2960 and 2890 cm^–1^ (vibrations ν (C–H) in CH_3_– methyl group), and the bands at 1460 and 1367 cm^−1^ (deformational vibrations in CH– group). However, no absorption bands related to organic matter were detected in Raman spectra of the same residue on sample 4.

Sample 5. At the high magnification (> 100 ×), the residues preserved on this sample (Fig. 6A-1) appear black in color (Fig. 6B). Like the FTIR spectra of the residues on samples 1, 3 and 4 described above, the FTIR spectrum of the residues on sample 5 (Fig. 6B) shows the bands at 2920 and 2850 cm^–1^ (vibrations ν (C–H) in CH_2_– methylene group), and the bands at 1460 and 1423 cm^−1^ (deformational vibrations in CH– group), which are typical to organic bitumen. Like sample 4, no bands related to organic matter were detected in Raman spectra of the residue on sample 5.

Additionally, the FTIR spectrum of the residue on sample 5 (Mezmaiskaya cave) shows the larger total number of absorption bands than the FTIR spectra of the residues on samples 1–4 (Saradj-Chuko grotto). This indicates a greater variety of inorganic compounds in the bitumen residue on sample 5 in comparison to the bitumen residues on samples 1–4. Also, Raman spectra indicate that three of the four analyzed bitumen residues from Saradj-Chuko grotto show the presence of absorption bands related to graphene/graphite organic compounds, which are absent in the bitumen sample from Mezmaiskaya (Table 2). These differences may indicate different origin sources of the bitumens identified on the lithic artefact from Mezmaiskaya cave in the north-western Caucasus and the four lithic artefacts from Saradj-Chuko grotto in the north-central Caucasus; the linear distance between the two MP sites is about 250 km. This assumption is confirmed by the SEM–EDS results.

#### SEM–EDS results

We used SEM–EDS to supplement the data obtained by FTIR and Raman spectroscopy by means of comparing the elemental composition of the archaeological residue (bitumen in our case) and the fresh areas (without the residue) representing the original material of the analyzed stone artefact. Molar ratios of main elements identified by SEM–EDS are represented in Table 3. The SEM–EDS analysis shows that unlike the areas without residue all bitumen residues on samples 1–5 are characterized by the highest value of carbon (C), the lowest value of silicon (Si), and the high C/Si value. These results indicate the different mineral composition of the residues, defined as organic bitumen on the basis of FTIR and Raman spectroscopy, and the original mineral material (flint or obsidian) of the analyzed lithic artefacts.

**Table 3:** Molar ratios of elements identified by SEM–EDS spectroscopy for areas with archaeological residue (bitumen) and fresh areas (without the residue) on samples 1–5.

**Sample 1**						
C	0.1670	0.0915	0.3685	0.0856	0.0963	**0.4618**
O	0.5920	0.2660	0.3416	0.6539	0.6815	**0.2401**
Si	0.2342	0.1716	0.1407	0.2502	0.1008	**0.0369**
C/Si	0.71	0.53	2.62	0.34	0.96	**12.50**
Si/O	0.40	0.65	0.41	0.38	0.15	**0.15**
**Sample 2**						
C	0.2490	0.1811	**0.7889**	**0.6534**		
O	0.4804	0.4142	**0.1862**	**0.3376**		
Si	0,0945	0,1940	**0,0142**	**0,0405**		
C/Si	2.63	0.93	**55.52**	**16.15**		
Si/O	0.20	0.47	**0.08**	**0.12**		
**Sample 3**						
C	0.2085	0.5572	0.1440	0.2053	0.4126	**0.4370**
O	0.5585	0.3277	0.6252	0.5131	0.3224	**0.2816**
Si	0.2330	0.1113	0.2241	0.0880	0.1215	**0.0237**
C/Si	0.89	5.01	0.64	2.33	3.39	**18.41**
Si/O	0.42	0.34	0.36	0.17	0.38	**0.08**
**Sample 4**						
C	0.2019	0.1372	0.4178	0.2144	0.1993	**0.3195**
O	0.5721	0.6227	0.2742	0.5628	0.5798	**0.4582**
Si	0.2115	0.2253	0.0867	0.1050	0.1025	**0.0199**
C/Si	0.95	0.61	4.82	2.04	1.94	**16.04**
Si/O	0.37	0.36	0.32	0.19	0.18	**0.04**
**Sample 5**						
C	0.1694	0.1955	**0.5404**			
O	0.6408	0.5928	**0.3958**			
Si	0.1705	0.1020	**0.0381**			
C/Si	0.99	1.92	**14.17**			
Si/O	0.27	0.17	**0.10**			

Areas with bitumen residue are in bold.

**Figure 7:**
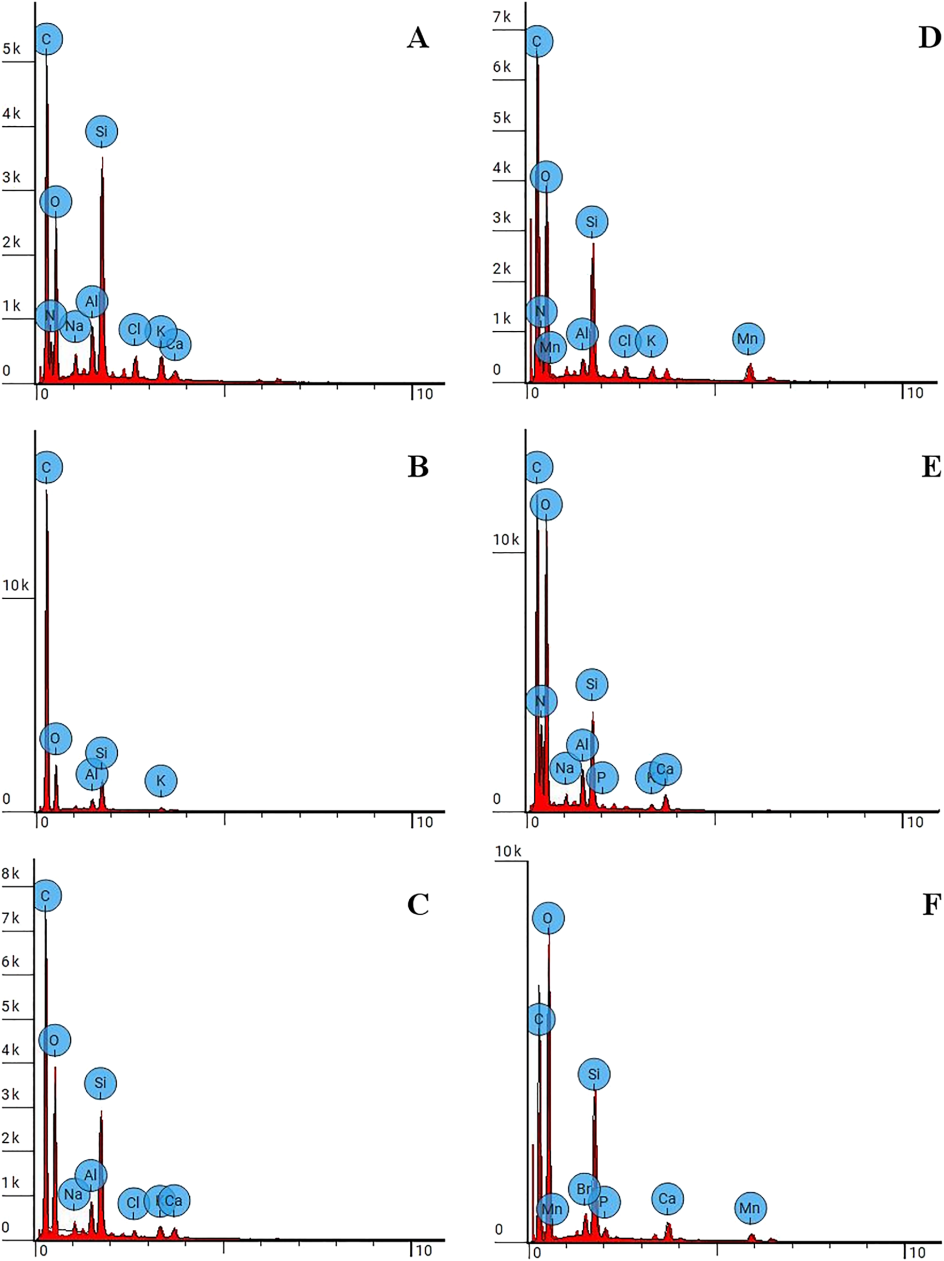
SEM–EDS spectra of bitumen residues identified on samples 1 (**A**), 2 (**B**, **C**), 3 (**D**), 4 (**E**), and 5 (**F**). Samples 1–4 are from Saradj-Chuko grotto and sample 5 is from Mezmaiskaya cave.

Most importantly, the ratio of C and O peaks, and the composition of other chemical elements in the bitumen samples 1–4 from Saradj-Chuko grotto are both different from that in the bitumen sample 5 from Mezmaiskaya cave (Fig. 7). Sample 5 shows the presence of Br not found in samples 1–4, but lacks Al, N, Na and K that are present in samples 1–4. These differences in elemental composition confirm the results of Raman spectroscopy that bitumens identified on stone artefacts from Mezmaiskaya cave and Saradj-Chuko grotto likely originate from different bitumen sources.

## Discussion and conclusions

Previously, we published results of microscope use-wear analyses of 62 lithic tools made of obsidian (52) and flint (10) from MP layer 6B at Saradj-Chuko grotto^28,37,38^. We identified the tools were used in a variety of activities, including as hunting weapons (spearheads), meat knives for butchering hunting prey, perforators or awls for hide-working, scrapers on wood or bone/antler, and stone retouchers.

Among the 13 tools from Saradj-Chuko grotto that we functionally identified as spearheads^37^, most have been typologically defined as convergent tools and convergent scrapers, and one as a Mousterian point. The identification of MP stone-tipped hunting spears has been made for the first time in the Caucasus. These tools had characteristic DIFs, such as transverse bending fracture with small spin-offs, and some had identifiable micro-traces of wear (smoothing and abrasion) resulted from mounting of the tool base on a wood shaft. We also defined a frequent supplementary use of the tools as bulb retouchers (seven of the 13 pieces). Also, micro-residues of a dark-brown or black substance, probably bitumen, were found on the surfaces of the basal parts of almost all of the tools. However, clear identification of these residues as the organic bitumen become possible only now, on the basis of the evidence presented in the current paper that confirms our previous assumption.

In the eastern North Caucasus (Terek River basin), besides Saradj-Chuko grotto, the only microscope use-wear analysis of five MP tools from layers 12–13, dated to Isotope Stages 4–5c (50–90 ka), at Weasel Cave (Myshtulagty Lagat) was published until now^39,40^. The microwear polishes analyzed on these archaeological specimens were interpreted as due to contact with meat, fresh and dry hide, bone, wood, as well as hafting, indicating that they were used as different instruments that served for one or two functions. The analyzed tools include atypical Mousterian point and Mousterian point. Both were defined as two-functional tools that have been used for butchering prey and planing wood, the first of them being used in a hafted mode.

Modern data indicates that the MP Neanderthals in the north-western Caucasus (Kuban River basin) were closely related culturally to the Neanderthal population bearing the Eastern Micoquian tradition in Eastern and Central Europe, while the MP Neanderthals that inhabited the north-central and north-eastern Caucasus (Terek River basin) were culturally similar with the Neanderthal population producing the Zagros Mousterian industry in the Lesser Caucasus and Zagros Mountains in Iran^27,28,41^. Cultural contacts between the Eastern Micoquian and Zagros Mousterian Neanderthal groups in the North Caucasus are assumed on the basis of finds of artefacts made from obsidian originating from the Zayukovo (Baksan) obsidian source in the north-central Caucasus at Mezmaiskaya cave and finds of typical Eastern Micoquian artefacts at Saradj-Chuko grotto^42^.

In the north-western Caucasus, the only known so far microscope traceological (use-wear) study of 131 MP lithic artefacts from Monasheskaya cave indicated that only 34 pieces had identifiable traces of use. The use-wear traces on these tools were interpreted as due to scraping or piercing hides, cutting meat and wood, and scraping wood and bone, as well as two pieces were defined as bulb retouchers. Among at least 8 convergent tools (five angled scrapers and three points) with identifiable use-wear traces, Shchelinsky^43^ defined scrapers/knives for hide-working, wood-working and butchering, as well as one angled scraper (or *dèjète* point) was defined as a probable projectile. No evidence of hafting or adhesive residues were identified on these tools.

Microscope use-wear studies are not yet done for stone artefacts from other Eastern Micoquian sites in the north-western Caucasus. In Mezmaiskaya cave, representing a reference Eastern Micoquian site in the region, recent microscope use-wear analysis and FTIR spectroscopy identified residues of bitumen and natural resin on a bone point served as a projectile tip, indicating the tool was likely mounted on a shaft, probably made from wood, and fixed using a glueing mastic composed of a mixture of bitumen and resin^44^. The data presented in this paper not only confirms this previous result but clearly indicate that the Eastern Micoquian Neanderthals in the north-western Caucasus used natural bitumen also for hafting stone tools.

In the South Caucasus, the only published so far microscope use-wear study of MP artefacts is the old analysis by S.A. Semenov^45^ of 12 Mousterian obsidian tools from Yerevan-1 cave in Armenia. Semenov identified the tools were mainly used for wood- and bone-working, and much more rarely for scraping, cutting and piercing hides, and butchering. The tools included three elongated Mousterian points and two Mousterian points, one of which represented the 'Yerevan-type' point with a truncated-faceted base. Semenov defined that most points were used as meat knives and also sporadically as awls, but not as projectiles. No evidence of hafting identified on these tools was reported.

Until recently, lithic residue studies were not performed in MP sites in the South Caucasus. The only residue analysis on 12 obsidian artifacts from MP deposits in Lusakert-1 cave (Armenia) published recently^46^ failed to identify any ancient organic substances, except probable chemical traces of proteins and animal fat of unknown provenance on two samples.

The objectives of this study is the functional characterization of MP tools from the Caucasus using comprehensive use-wear and residue analysis basing on modern microscopic and spetroscopic methods, that was made for the first time in this region. Using reflected light stereomicroscopy, we defined that four of the five analyzed tools (one side-scraper and three convergent scrapers) were used as meat knives, but all convergent scrapers also have DIFs indicating that their tips were broken due to projectile impact. This suggests the convergent scrapers were used originally as a projectile tip and secondary as a meat knife. One tool (Mousterian point) was functionally identified as a projectile tip, and probably served as a spearhead. Also, bases of all five tools bear diagnostic hafting traces, such as wood/antler hafting polish, and also exhibit micro-residues of a dark brown/black substance, which was preliminary identified as bitumen. The characteristics and location of DHTs and possible bitumen residues suggest that the bases of these tools were hafted, probably in a wooden haft or shaft using bitumen.

Furthermore, we for the first time documented the use of organic bitumen for hafting MP tools in the Caucasus. The bitumen was identified and its elemental composition was characterized using three complementary methods, including FTIR, Raman and SEM–EDS spectroscopy. The combination of these methods allowed us to precisely determine organic and inorganic substances preserved on the surfaces of artefacts, and draw conclusion about the use of bitumen-based adhesives for hafting stone tools by MP Neanderthals in the North Caucasus. The results agree with the previous residue analyses, which indicated that MP hominins were making hafted spear points and other tools using natural hafting adhesives, such as bitumen and birch-bark pitch^20,23,47–49^.

Our study unequivocally indicates the use of bitumen for hafting lithic tools in at least two different MP cultural contexts associated with the Neanderthals in the region, the Eastern Micoquian in the north-western Caucasus (Mezmaiskaya cave) and the Zagros Mousterian in the north-central Caucasus (Saradj-Chuko grotto). Moreover, the Raman spectroscopy and SEM–EDS data indicate that bitumens identified on stone artefacts from Mezmaiskaya cave and Saradj-Chuko grotto likely originate from two different bitumen sources. From a methodological point of view, this integrated functional study of MP (Mousterian) lithic tools emphasizes a high reliability of the functional interpretation of Paleolithic lithic artefacts that is based on the integration of use-wear traces, morphological features of lithic artefacts, physicochemical characterization of residues, and the distribution patterns of various functional modifications and residues on the analyzed artefacts.

## Materials and methods

We studied five lithic tools found in layers 6A and 6B at Saradj-Chuko grotto, and in Layer 2B-3 at Mezmaiskaya cave (Table 1). These lithic artefacts were recovered from modern excavations, and show a good preservation of both the organic and inorganic residues that adhered to their surfaces, and traces of wear from use and hafting. All five artifacts that were used for this study have no traces indicating that their surfaces have been altered due to post-depositional processes. The artifacts are not patinated or polished due to soil polish or other natural post-depositional alterations, and their surfaces are fresh. The only exception is sample 5, which is covered by calcite-carbonate crust on some areas of the tool surface, which looks fresh in all areas not covered with the crust.

### Archaeological sites

Mezmaiskaya Cave is located 1310 m above sea level (asl), in a small tributary (the Sukhoi Kurdjips River) of the Kurdjips River (itself a tributary of the Belaya River, Kuban River basin), in the Azish-Tau ridge (Lago-Naki highland), about 50 km south of the city of Maikop, in the north-western Caucasus, Russia (Fig. 1). The cave is formed in the Upper Jurassic dolomite limestone cliff about 20 m in height, and is situated about 100 m above the Kurdjips River level. It is more than 500 m^2^ (15–17 m in width and about 35 m in length), up to 10 m in height in the entrance, and faces southwest. In the interior of the cave, there is a chamber with a relatively horizontal floor while near the entrance the modern surface of the cave deposits is gently sloping outside. Since 1987, when L. Golovanova started excavations on the site, about 80 m^2^ have been carefully excavated to a maximum depth of 5 m.

Mezmaiskaya cave preserves a finely layered sedimentary succession of Late Pleistocene and Holocene deposits. The basal Pleistocene strata (4–7)—excavated only in a test pit—contained no archaeological material. So far, 6 Holocene and 20 Late Pleistocene strata have been identified over the excavation area, including seven Middle Paleolithic levels (2, 2A, 2B-1, 2B-2, 2B-3, 2B-4, and 3, from top to bottom) with ESR dates between ca. 70–40 ka BP^50,51^; six Upper Paleolithic and two Epipaleolithic levels dating to ca. 39–25 ka cal BP and 17.5–12.5 ka cal BP respectively^52^; and six post-Paleolithic levels dating from the Holocene.

Mezmaiskaya cave is widely known as a reference late Middle Paleolithic Micoquian occupation^27,41,53–58^, which has yielded three well-preserved Neanderthal fossils. They include the skeleton of a Neanderthal neonate (Mez 1), discovered in 1993 in the oldest Middle Paleolithic Layer 3; an isolated permanent tooth (Mez 3), which was found later, also in Layer 3; and skull fragments of a Neanderthal child (Mez 2), which were found in 1994 in Layer 2, the uppermost Middle Paleolithic level^59–63^.

Saradj-Chuko Grotto is located 935–940 m asl, in the Saradj-Chuko (or Fanduko) River, which is a small left tributary of the Kishpek River (itself a tributary of the Baksan River, Terek River basin), approximately 20 km northwest of the city of Nalchik (Kabardino-Balkaria republic), in the Elbrus region of the north-central Caucasus, Russia (Fig. 1). The cave is formed in the Pliocene acidic volcanites (ingimbrites and tuffs), and is situated about 26 m above the Saradj-Chuko River level. It is more than 300 m^2^ (up to 22 m in width and about 20 m in length), up to 6 m in height in the domelike entrance, and faces southeast.

Since 2016, when E. Doronicheva started excavations on the site, about 46 m^2^ have been carefully excavated in 2017–2019^28,64,65^. The cave stratigraphic sequence is about 1–1.5 m thick. The dense basal deposit (layer 7) is an archaeologically sterile stratum, composed of ignimbrite and tuff slabs and fine-grained sediments. The overlying layers 6B, 6A and 3, with optically stimulated luminescence (OSL) dates from 92 to 41 ka BP, have yielded over 11,600 stone artifacts and numerous animal remains. The Middle Paleolithic sequence is capped by layer 2 with rare artefacts and Holocene deposits (layers 1, 1A–1C). Saradj-Chuko grotto is a reference Middle Paleolithic occupation, which produced the only Mousterian obsidian industry known in the North Caucasus and the first laminar industry attributed to Zagros Mousterian in this region.

### Methods

#### Use-wear analysis

For the traceological (use-wear) analysis we applied reflected light microscopy, using a MS-2ZOOM stereomicroscope (LOMO, Russia) with magnification up to 80 × and a MSP-2 stereomicroscope (LOMO, Russia) with magnification up to 160x, and TOUPCAM video-eyepiece and MS-12 digital camera. The analysis was made using the facilities of the ANO Laboratory of Prehistory, St. Petersburg (Russia). The identification and interpretation of DIFs, DHTs, and use-wear micro-traces on the analysed lithic artefacts is based both on the traceological method of use-wear analysis and diagnostic criteria developed in the Laboratory for Experimental-Traceological Studies in the Institute for the History of Material Culture of the Russian Academy of Sciences, St. Petersburg^66–69^. We also apply criteria defined in the scientific literature^29,30,70,71^. The DIFs indicating the potential use of the lithic tool as a tip of a composite projectile are defined based on the criteria developed by other researchers^6,8,11–15,72^.

#### Lithic residue analyses

The elementary composition of the analyzed archaeological residues has been obtained by means of scanning electron microscopy (SEM) with energy dispersive spectroscopy (EDS), while their chemical structure was investigated using Fourier transform infra-red (FTIR) spectroscopy and Raman spectroscopy. FTIR and Raman spectroscopy are mutually complementary spectroscopy methods, which together provide the most complete analytical results for identification of chemical substances and compounds. These methods were used for the identifcation of bitumen on all archaeological samples in our study. The analyses were made using the facilities of the Shared Research Center “Analytical center of deep oil processing and petrochemistry” in the A.V. Topchiev Institute of Petrochemical Synthesis (TIPS) Russian Academy of Sciences, Moscow (Russia).

The micro-samples for FTIR spectroscopy were taken with a preparative needle from dark colored substances preserved on the surfaces of the analyzed artifacts. FTIR spectra were recorded on a Bruker IFS 66 v/s FTIR spectrometer with spectral range coverage 4000–500 cm^−1^, using the Bruker OPUS software. Raman and SEM–EDS spectroscopy were made directly on the surfaces of archaeological samples. Raman spectra were recorded on a Bruker Senterra II confocal Raman microscope, in full spectral range with 4 cm^−1^ resolution. The spectra were obtained from different parts of the study area on the analyzed samples. SEM–EDS spectroscopy was performed on a Phenom XL G2 desktop scanning electron microscope (Thermo Fisher Scientific) equipped with an EDS spectroscope, with accelerating voltage of 15 kV and vacuum pressure of 10 Pa. The SEM–EDS spectra were acquired using the built-in elemental identification software for the automated peak analysis. For the greater reliability of the SEM–EDS results, the analysis was carried out in 3–4 points and areas (without the residue and with the residue) on each archaeological sample. For each sample we calculated molar ratios of main chemical elements identified by SEM–EDS.

The good preservation of the analyzed lithic artefacts allowed us to identify use-wear traces and residues on all archaeological samples. Various articles focused on the characterization and identification of common materials found in various applications^32–34,73–76^ and especially on the study of residues preserved on Paleolithic stone tools [e.g.^9,49,77–81^] established the methodological framework for examination of archaeological residues in our study. The absorption bands corresponding to specific bitumen bands were defined after^32–34,73–75^. Band assignments for Raman peaks were made based on^36^.

## Data Availability

The datasets used and/or analysed during the current study available from the corresponding author on reasonable request.
